# Troublesome Heterotopic Ossification after Central Nervous System Damage: A Survey of 570 Surgeries

**DOI:** 10.1371/journal.pone.0016632

**Published:** 2011-01-31

**Authors:** François Genêt, Claire Jourdan, Alexis Schnitzler, Christine Lautridou, Didier Guillemot, Thierry Judet, Serge Poiraudeau, Philippe Denormandie

**Affiliations:** 1 Service de Médecine Physique et de Réadaptation, Hôpital Raymond Poincaré, Garches, France; 2 Service de Chirurgie Orthopédique et Traumatologique, Hôpital Raymond Poincaré, Garches, France; 3 Unité Fonctionnelle de Santé Publique, Hôpital Raymond Poincaré, Garches, France; 4 Service de Médecine Physique et de Réadaptation, Hôpital Cochin, Paris, France; Brigham and Women's Hospital, Harvard Medical School, United States of America

## Abstract

**Background:**

Heterotopic ossification (HO) is a frequent complication after central nervous system (CNS) damage but has seldom been studied. We aimed to investigate features of HO for the first time in a large sample and the rate of early recurrence of HO in terms of the time of surgery.

**Methodology/Principal Findings:**

We retrospectively analyzed data from an anonymous prospective survey of patients undergoing surgery between May 1993 and November 2009 in our institution for troublesome HO related to acquired neurological disease. Demographic and HO characteristics and neurological etiologies were recorded. For 357 consecutive patients, we collected data on 539 first surgeries for HO (129 surgeries for multiple sites). During the follow-up, recurrences requiring another surgery appeared in 31 cases (5.8% [31/539]; 95% confidence interval [CI]: 3.8%–7.8%; 27 patients). Most HO requiring surgery occurred after traumatic brain injury (199 patients [55.7%]), then spinal cord injury (86 [24.0%]), stroke (42 [11.8%]) and cerebral anoxia (30 [8.6%]). The hip was the primary site of HO (328 [60.9%]), then the elbow (115 [21.3%]), knee (77 [14.3%]) and shoulder (19 [3.5%]). For all patients, 181 of the surgeries were performed within the first year after the CNS damage, without recurrence of HO. Recurrence was not associated with etiology (p = 0.46), sex (p = 1.00), age at CNS damage (p = 0.2), multisite localization (p = 0.34), or delay to surgery (p = 0.7).

**Conclusions/Significance:**

In patients with CNS damage, troublesome HO and recurrence occurs most frequently after traumatic brain injury and appears frequently in the hip and elbow. Early surgery for HO is not a factor of recurrence.

## Introduction

Heterotopic ossification (HO) is defined as the formation within the soft tissues of abnormal, ectopic lamellar bone containing bone marrow [1], [2], [3], [4]. It has 3 etiologies: 1) trauma (fractures, dislocations, post-surgery, burns), 2) genetic (fibrodysplasia ossificans progressiva (FOP), progressive osseous heteroplasia and Albright hereditary osteodystrophy), and 3) neurologic (mainly spinal cord injury [SCI] and traumatic brain injury [TBI]) [1], [5], [6].

In patients with central nervous system (CNS) damage, HO is a frequent complication, ranging from 11% to 76% of cases depending on the etiology and the study because of varied diagnostic criteria [7], [8], [9]. Symptomatic HO develops in approximately 10% of patients with TBI [10], [11], [12]. In SCI, the HO frequency varies from 5% to 60% depending on the study and whether the diagnosis is based on clinical symptoms or standard radiography results [13], [14], [15], [16].

The etiopathogenesis of HO is poorly understood [2]. CNS damage is believed to activate local factors such as bone morphogenic protein or systemic factors such as prostaglandin E2, or both [1], [4], [6]. These factors could induce bone-forming mesenchymal cells to differentiate to osteoblasts in the periphery of the muscle and stimulate the formation of bone[1], [4], [12], [17]. Another potential mechanism is the disruption of joint proprioception after neurologic damage, thus changing the relationship among the different peri-articular tissues [18]. For patients with TBI, osteogenic blood factors have been suggested[12], [17].

For patients with CNS damage, HO causes pain, inflammation and loss of range of motion (ROM) as the joint gradually becomes ankylosed [4], [5], [12], [19], [20]. The condition may have major repercussions on function, with, in many cases, loss of independence [2], [6]. Currently, the only effective treatment is surgery [4], [5], [19], [20], [21]. Indications for surgery have changed recently [4]. Until recently, surgery was delayed until the HO was fully formed [4], [21], [22], [23], [24], and studies of small samples of patients (analyzed by reviews) have suggested that the rate of recurrence is not affected by HO maturity [4], [25], [26]. Indications for surgery relate to vascular or neurological involvement, effect on function, hygiene (e.g., access to the perineum) and pain [5], [20], [26]. Surgery can be performed as soon as co-morbidity factors are under control, even in patients with major neurologic damage due to the original abnormality (e.g., TBI) [2], [26], [27], [28].

Only a few studies have investigated the epidemiological features of HO (incidence, recurrence, localization) in adult patients with CNS lesions [25]. Furthermore, these studies included few patients [2], [10], [13], [26], [29]. The most important series was published by Garland et al., in 1980; the incidence of HO was 11% in a cohort of 496 patients with TBI in a physical medicine and rehabilitation unit [10]. In 2005, Fuller et al. reported on 17 patients with 22 knee HO in a retrospective analysis [19], Simonsen et al. in 2007, reported on 13 patients with HO in 21 locations in a prospective cohort of 114 patients with TBI [29], and Melamed et al. reported on 12 excisions for HO in 9 patients with TBI [26].

We aimed to investigate a neuro-orthopedic complication — HO requiring surgery – in a large cohort of patients with CNS damage admitted to an orthopedic and trauma hospital ward. This unit is part of a tertiary-care teaching hospital specialized in medical treatment, surgery and rehabilitation for motor handicap. We also investigated the rate of recurrence of HO in terms of time to surgery. Results from this large survey might help establish recommendations for treating HO after CNS damage.

## Methods

### Objectives

The objectives are to investigate features of HO for the first time in a large sample of patients and notably to assess if the time of surgery is associated with the risk of early recurrence of HO.

### Participants

This prospective data survey involved consecutive patients undergoing surgery between May 1993 and November 2009 for HO of a joint after CNS injury. Patients with previous removal of HO (before their inclusion in the study) who presented recurrence or could not be followed up by the surgeon or the physical medicine and rehabilitation physician for a minimum of 6 months were excluded, as were those without an initial neurologic aetiology.

### Description of Procedures or Investigations undertaken

Patients were referred for a specialized neuro-orthopedic consultation for troublesome HO. Indications for surgery were loss of ROM with functional repercussion, ankylosed joint, and nerve or vessel compression. The surgery and immediate post-operative assessment were performed by the same surgeon.

The following data were collected during consultation by questionnaire and medical records : sex; etiology of the CNS damage (ischemia or hemorrhage for stroke; tetraplegia or paraplegia for SCI, with lesion level; brain-associated lesion [TBI or cerebral anoxia (CA)] and American Spinal Injury Association [ASIA] score [30]); age at CNS damage and at surgery; delay from neurologic trauma to surgery; affected joints; type of HO for the surgical approach of Garland (for incision) [7] (from radiography and computed tomography [CT] scanning: anterior, posterior, internal [anterior and posterior], external [anterior and posterior], and encircling); area of residence (Paris area [Ile de France], near Ile de France, between 200 and 400 km from Ile de France, more than 400 km from Ile de France, and foreign countries and French overseas departments and territories); and follow-up (last consultation, months). Finally, data were collected on complications, especially sepsis and HO recurrence. Several patients had received prophylactic treatment for HO, such as a nonsteroidal anti-inflammatory drug (for pain) into their upstream structure. Radiation therapy was rarely used by the upstream units.

A standardised surgical approach was used for each location of the HO. The surgical goal was resection of the necessary amount of bone to allow for restoration of motion in all planes. Postoperatively, gentle mobilization was started on the second day, and progressed as tolerated. A non-steroidal anti-inflammatory agent, ketoprofene, was given for 10 days after surgery. Neither radiation therapy (RT) nor indomethacin was used after surgery. RT was never carried out after surgery mainly because the post-operative management of such a treatment is too complicated for these kinds of patients. RT was sometimes administered before surgery but only when recurrence occurred (14/31 cases). The main reason was that we are not aware of any evidence demonstrating the efficacy of RT for HO after central nervous system damage. The recurrence rate was very low and well distributed across the 16 years of this study. As there are no recommendations for patients with neurological lesions, we used the same RT protocol as for hip arthroplasty in the patients who received pre-operative RT (4 hours before surgery, 7 to 10 Gy) [31].

Patients were followed up in the rehabilitation unit of the same institution for surgery if they lived locally or by medical consultation if they lived far away. Each patient underwent regular clinical and radiography examinations; they were hospitalized in a surgical care unit for about 1 week, then received regular consultations in rehabilitation units (inpatient care followed by outpatient care for a minimum of 1 year). All patients received intravenous peri-operative antibiotic prophylaxis and anticoagulation medication.

### Ethics

The study was approved by the local institutional review board. It was a non-interventional study with usual procedures and without additional procedures (diagnosis or medical supervision). In France, patient consent is not needed for such an anonymous retrospective data analysis. We confirm that the named institutional review board specifically waived the need for consent for this study [Comité de Protection des Personnes Ile de France XI Pavillon Jacques Courtois - 2ème étage 20, rue Armagis 78105 Saint Germain en Laye Cedex. tél : 01.39.27.42.58 - fax : 01.39.27.49.01 mail : cppidf11@chi-poissy-st-germain.fr"]

### Statistical methods

Statistical analysis involved use of SAS® v9.1 (SAS Inst., Cary, NC). Data are reported as median, interquartile ranges (IQRs) and numbers and percentages. The chi-square test was used to compare normally distributed qualitative variables and the Fisher test for no normally distributed data. ANOVA was used for analysis of continuous variables, and if significant, the Student *t* test was used to compare groups with the nearest values. All p values were two tailed, and a p<0.05 was considered statistically significant.

## Results

### Demographic data (Table 1)

**Table 1:** Demographic Data for Patients Undergoing Surgery for Heterotopic Ossification After Central Nervous System (CNS) Damage.

Patient parameters	TBIn = 199(55.7)	Stroken = 42(11.8)	SCIn = 86(24.1)	CAn = 30(8.4)	Totaln = 357(100)
Male (%)	159(79.9)	26(61.9)	81(94.2)	21(70.0)	287(80.4)
Age at time of CNS damage (yr)medianinterquartile range	30.623.6–38.8	45.737.9–51.5	27.121.3–34.7	44.932.2–48.3	32.424.6–43.3
Age at time of surgery (yr)Medianinterquartile range	32.226.2–41.2	49.740.5–54.3	34.727.1–42.7	46.534.3–49.3	35.427.6–46.7
Delay from CNS damage to first surgery (months)Medianinterquartile range	13.18.3–29.0	15.29.5–34.7	24.113.4–72.4	12.79.5–21.0	15.79.2–37.5

HO: Heterotopic Ossification; TBI: traumatic Brain Injury; SCI: Spinal Cord Injury; CA: Cerebral Anoxia; CNS: Central Nervous System.

**Figure 1:**
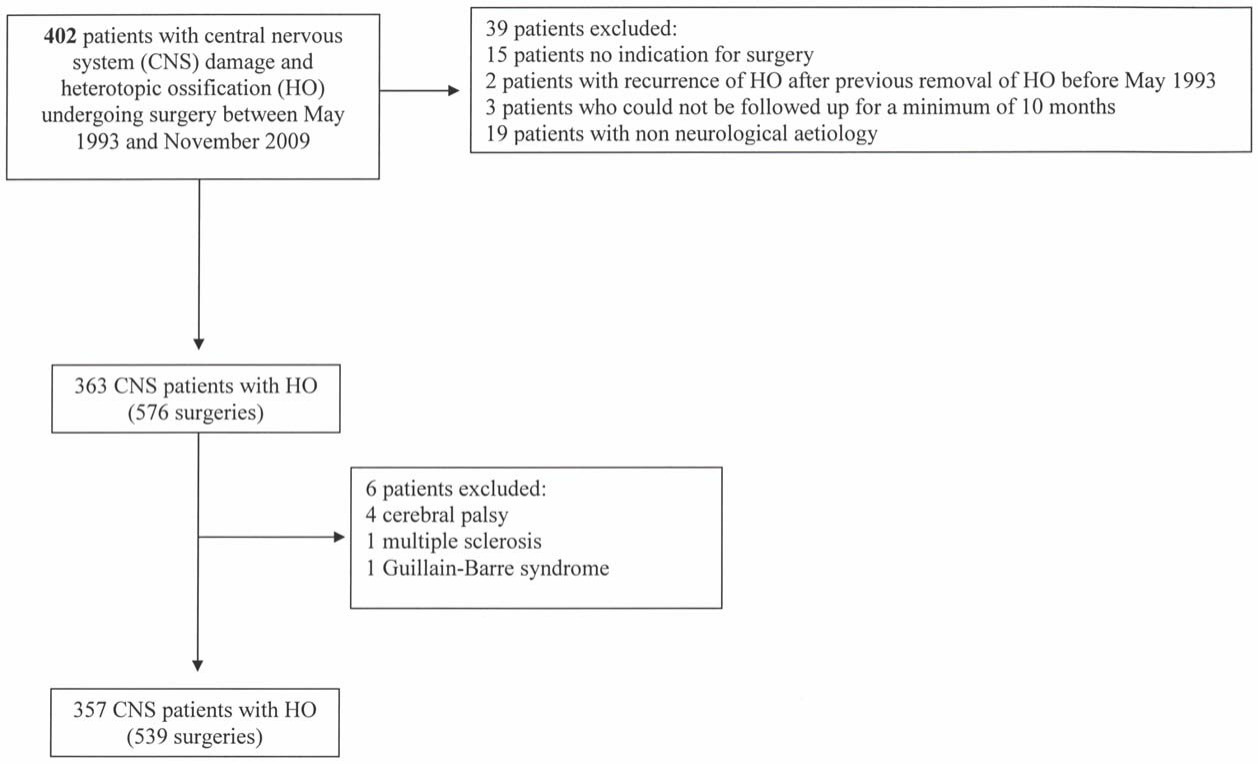
Flow chart of the patients' selected files.

Of 402 consecutive patients undergoing surgery for HO between May 1993 and November 2009, 363 with neurologic damage met our inclusion criteria (Fig. 1). Data for the few patients with cerebral palsy (4 patients), multiple sclerosis (1 patient) and Guillain-Barré syndrome (1 patient) were excluded from the analysis. Thus, we analyzed the data for 357 patients (70 females) who underwent 570 surgeries: 539 (94.2%) were first surgeries (multiple sites for 129 patients: 2 surgeries for 97 patients, 3 for 18; 4 for 9, 5 for 3 and 6 for 2) and 31 were for recurrences for a rate of recurrence of 5.8% (95% confidence interval [CI]: 3.8–7.8%).

The median delay from neurological trauma to the first surgery was 15.7 months (IQR 9.2 to 37.5 months) (Table 1), and the median delay with multi-site HO was 16.7 months (IQR 9.9 to 40.0 months). Most cases of HO were related to TBI (199 patients [55.7%], 304 surgeries [56.4%]), then SCI (86 patients [24.1%]; 56 paraplegia and 30 tetraplegia; 129 surgeries [23.9%]), stroke (42 patients [11.8%], 10 ischemia and 32 hemorrhage; 55 surgeries [10.2%]) and CA (30 patients [8.6%]; 51 surgeries [9.5%]). The median follow-up by the surgeon was 6.9 months (IQR 5.7 to 19.4 months).

The presence of multiple sites of HO occurred more frequently in patients with CA (16/30; 14 patients with 2 locations, 1 patient with 3 locations and 1 patient with 6 locations) and TBI (74/199; 53 patients with 2 locations, 14 with 3 locations, 4 with 4 locations and 3 with 5 locations) than with SCI (29/86; 14/56 with paraplegia, 15/30 with tetraplegia; 22 with 2 locations, 2 with 3 locations, 4 with 4 locations and 1 patient with 6 locations) and stroke (10/42; 8 patients with 2 locations, 1 patient with 3 locations and 1 patient with 4 locations).

### HO surgery side effects

The main side effects were sepsis (16; 3.0%) and HO recurrence (31; 5.8%). All patients with sepsis underwent secondary surgery. Sepsis occurred mainly after SCI (12), then TBI (3) and stroke (1). All patients with HO recurrence underwent secondary surgery. Recurrences occurred after TBI (16), stroke (2), SCI (10), and CA (3). In total, 27 patients (15 with multisite HO) had 1 recurrence and 4 patients (all with multisite HO) had 2 recurrences on 2 different joints. HO recurrence concerned only the hip (25 recurrences) and the elbow (6). Recurrence was more frequent for patients with multisite HO (15/27; 55.6%) than for all patients (127/357; 35.6%). For the 15 patients with multisite HO, recurrence never appeared after the first surgery but after a later surgery on a different site. For 181 surgeries performed within the first year after the CNS damage, no recurrence was reported at 6-month follow-up.

### HO sites (Table 2)

**Table 2:** Sites of Heterotopic Ossification (HO) in Patients Undergoing Surgery After Central Nervous System Damage.

Location of HO	TBIn = 304	Stroken = 55	SCIn = 129	CAn = 51	Totaln = 539
**Hip – no. (%)**	163 (53.6)	40 (72.7)	96 (74.4)	29 (56.9)	328 (60.9)
**Knee – no. (%)**	43 (14.1)	8 (14.6)	19 (14.7)	7 (13.7)	77 (14.3)
**Shoulder – no. (%)**	13 (4.3)	1 (1.8)	2 (1.6)	3 (5.9)	19 (3.5)
**Elbow – no. (%)**	85 (28.0)	6 (10.9)	12 (9.3)	12 (23.5)	115 (21.3)
**Total – no. (%)**	304 (56.4)	55 (10.2)	129 (23.9)	51 (9.5)	539 (100)

Data are for Number of First Surgeries Performed.

TBI: traumatic brain injury; SCI: spinal cord injury; CA: cerebral anoxia; CNS: central nervous system.

For all first surgeries (all etiologies combined), the primary site for HO was the hip joint (328/539; 60.9%). Hip HO occurred most frequently with SCI (96/129; 74.4%) and stroke (40/55; 72.7%). Elbow HO was the next most affected joint (115/539, 21.3%), then the knee (77/539; 14.3%) and shoulder (19/539; 3.5%). Elbow HO occurred most frequently in patients with TBI (85/304; 28.0%) and CA (12/51; 23.5%). Knee HO occurred most frequently in patients with SCI (19/129; 14.7%; 7 for paraplegia, 2 bilateral; and 6 for tetraplegia, 4 bilateral), stroke (8/55; 14.1%) and TBI (43/304; 14.1%). The ratio of knee to hip HO (81.0%) and shoulder to elbow HO (85.8%) was similar for all etiologies.

### Lower-limb HO sites (Table 3)

**Table 3:** Sites of Heterotopic Ossification of Patients Undergoing Surgery for Central Nervous System Damage. Data are for Number of First Surgeries Performed.

Site	Hip					*Knee*				
	TBIn = 304	Stroken = 55	SCIn = 129	CAn = 51	Totaln = 539	TBIn = 304	Stroken = 55	SCIn = 129	CAn = 51	Totaln = 539
**Anterior – no. (%)**	27 (16.9)	9 (23.1)	46 (50.0)	6 (23.1)	88 (27.8)	2 (4.9)	0 (0.0)	1 (5.3)	0 (0.0)	3 (4.0)
**Posterior – no. (%)**	29 (18.1)	1 (2.6)	12 (13.0)	7 (26.9)	49 (15.5)	4 (9.8)	0 (0.0)	0 (0.0)	0 (0,0)	4 (5.3)
**Internal – no. (%)**	56 (35.0)	20 (51.3)	19 (20.7)	7 (26.9)	102 (32.2)	31 (75.5)	7 (87.5)	16 (84.1)	4 (57.1)	58 (77.3)
**External – no. (%)**	28 (17.5)	7 (17.9)	3 (3.3)	2 (7.7)	40 (12.6)	2 (4.9)	1 (12.5)	1 (5.3)	1 (14.3)	5 (6.7)
**Encircling – no. (%)**	20 (12.5)	2 (5.1)	12 (13.0)	4 (15.4)	38 (11.9)	2 (4.9)	0 (0.0)	1 (5.3)	2 (28.6)	5 (6.7)
**Total**	160 (50.5)	39 (12.3)	92 (29.0)	26 (8.2)	317 (100)	41 (54.7)	8 (10.7)	19 (25.3)	7 (9.3)	75(100)

Site	Shoulder					*Elbow*				
	TBIn = 304	Stroken = 55	SCIn = 129	CAn = 51	Totaln = 539	TBIn = 304	Stroken = 55	SCIn = 129	CAn = 51	Totaln = 539
**Anterior – no. (%)**	0 (0.0)	0 (0.0)	2 (100.0)	0 (0.0)	2 (11.8)	3 (3.8)	0 (0.0)	0 (0.0)	2 (16.7)	5 (4.6)
**Posterior – no. (%)**	4 (40.0)	0 (0.0)	0 (0.0)	0 (0.0)	4 (23.5)	22 (27.5)	0 (0.0)	3 (25.0)	3 (25.0)	28 (25.4)
**Internal – no. (%)**	4 (40.0)	1 (100.0)	0 (0.0)	1 (33.3)	6 (41.2)	43 (53.7)	6 (100.0)	9 (75.0)	5 (41.7)	63 (57.3)
**External – no. (%)**	0 (0.0)	0 (0.0)	0 (0.0)	0 (0.0)	0 (0.0)	12 (15.0)	0 (0.0)	0 (0.0)	0 (0.0)	12 (10.9)
**Encircling – no. (%)**	2 (20.0)	0 (0.0)	0 (0.0)	2 (66.7)	4 (23.5)	0 (0.0)	0 (0.0)	0 (0.0)	2 (16.7)	2 (1.8)
**Total**	10 (62.5)	1 (6.3)	2 (12.5)	3 (18.7)	17 (100.0)	80 (72.7)	6 (5.5)	12 (10.9)	12 (10.9)	110 (100.0)

HO in the anterior and internal hip represented 60.0% of the total sites. This incidence was highest for patients with stroke (74.4%), then SCI (70.7%), TBI (51.9%) and CA (50.0%). The principle site of knee HO was internal (77.3%). This incidence was highest for patients with stroke (87.5%), then SCI (84.1%), TBI (75.5%) and CA (57.1%).

### Upper-limb HO sites (Table 3)

The main site of elbow HO was posterior and internal (82.7%). Similar to hip HO, this incidence was highest for patients with SCI (100.0%; all tetraplegia and 1 paraplegia with associated elbow fracture) and stroke (100.0%), then TBI (81.2%) and CA (66.7%). Only 5 patients with paraplegia exhibited HO in the upper limbs: 4 had an associated brain injury (2 with HO in shoulders and 2 elbows) and one a medical SCI with HO after elbow fracture. Only 19 occurrences of HO (3.5%) were in the shoulder joint, and the main sites were internal (41.2%), posterior (23.5%) and encircling (23.5%).

### Univariate analysis of data for patients (Tables 4)

**Table 4:** Univariate Analysis of Data for Patients (n = 357).

Variables	*p (F statistic)*	*p*
**Etiology**		
**Delay until first surgery**	<0.01* (11.46)	
**Delay until first surgery for SCI and stroke**	<0.01* (7.38)	
**Multisite HO**		0.1
**Recurrence**		
**Multisite HO**		0.34
**Sex**		1.00
**Etiology**		0.46
SCI = spinal cord injury; HO = heterotopic ossification		

HO: heterotopic ossification; TBI: traumatic brain injury; SCI: spinal cord injury; CA: cerebral anoxia; CNS: central nervous system.

When analyzing data for patients (n = 357), we found a significant association between etiology and delay until first surgery (F = 11.5; p<0.01). The shortest delay was observed for CA (12.7 months; IQR 9.5 to 21.0 months), then stroke, TBI and SCI. We found a significant difference between delay until first surgery for SCI (the longest delay) and for stroke (the nearest from SCI) (F = 7.38; p<0.01). We suggest that differences between SCI and the other 2 etiologies (TBI and CA) are significant also. We did not find an association of etiology and multiple-site HO (p = 0.1). Recurrence was not associated with etiology (p = 0.46), sex (p = 1.00), or multisite HO (p = 0.34).

### Univariate analysis of time from CNS damage related to HO recurrence (Table 5)

**Table 5:** Univariate Analysis of Time from Central Nervous System Damage Related to Heterotropic Ossification Recurrence.

Variables	*P (F statistic)*	With recurrence	Without recurrence
**Data for Patients (n = 357)**			
**Delay from CNS damage to surgery – mo.**	0.7 (0.13)	21.5 (9.0; 34.1)	15.6 (9.2; 37.5)
**Age at CNS damage – years**	0.2 (1.65)	30.0 (21.9; 36.6)	32.5 (24.7; 43.4)
**Data for Surgeries (n = 539)**			
**Delay from CNS damage to surgery – mo.**	0.13 (2.35)	28.7 (12.0; 59.1)	16.4 (9.9; 37.9)
Data are median (interquartile range) CNS = central nervous system			

When analyzing data for patients (n = 357), recurrence was not associated with age at CNS damage (F = 1.65; p = 0.20) or delay from CNS damage to first surgery (months) (F = 0.13; p = 0.7). For all surgeries, (including multisite HO), recurrence was not associated with delay from CNS damage to surgery (months) (F = 2.35; p = 0.13).

## Discussion

HO is a frequent complication after CNS damage. This survey of a large sample of patients with CNS damage revealed that most HO requiring surgery occurred after TBI, then SCI, stroke and CA. Multi-site HO was most frequently due to CA and TBI. For all patients, the hip was the primary site of damage, then the elbow, knee and shoulder. The median time from CNS damage to surgery for all etiologies was 15.7 months (IQR 9.2 to 37.5 months), which was shorter than that for SCI (24.1 months; IQR 13.4 to 72.4 months). For all patients, 181 of the surgeries were performed within the first year after the CNS damage, without recurrence at 6-month follow-up. Recurrence was not associated with etiology, sex, age at CNS damage, multisite HO, or delay from CNS damage to surgery, whether analyzing data for patients or surgeries. No association was found between etiology and multisite HO or articulation location of HO. These results suggest that surgery could be proposed as soon as HO becomes troublesome.

In our survey, HO was mainly observed in 4 etiologies and very rarely in other disorders such as multiple sclerosis (1), cerebral palsy (4), or Guillain-Barré syndrome (1). Our observations are unlikely due to recruitment bias because our institution, in charge of the National Adult CP Network, is the main site for HO management in France. This is suggested by our geographical recruitment [Ile de France 220 (63.9%); near Ile de France 41 (11.9%); between 200 and 400 km from Ile de France 22 (6.4%); more than 400 km from Ile de France 23 (6.7%); and foreign countries or French overseas departments and territories 38 (11.1%)]. Better risk factors for HO have been reported to be related to coma severity, coma length, presence of diffuse axonal lesions, spasticity, systemic infection, and overall dysautonomia, which is a high predictive factor of HO [11], [25]. These risk factors may explain why HO was observed mainly for patients with CA, TBI, SCI and stroke. Furthermore, notably after TBI, neurological repercussions affect the whole organism and probably also bone hormonal control [32].

As suggested by Garland et al., we found that the main site of HO was the hip (60.9%), then the elbow (21.3%), knee (14.3%) and shoulder (3.5%) [10]. The high incidence in the hip may be linked to HO in this joint having large repercussions on function (e.g., ability to sit, lie or stand). The high incidence in the elbow may be linked to the frequency of ulnar nerve compression, which is a strong indication for surgery. Furthermore, HO often occurred below the level of the CNS lesion in patients with SCI, which may explain the high incidence of lower-limb HO in this group. For the hip, the sites of HO seem to depend on the abnormality: the most frequent site for SCI was anterior (51.1%) but antero-medial for stroke (43.6%). This finding could reflect joint-related constraints, which differ according to muscle control. However, this hypothesis needs to be confirmed with further studies. For all etiologies, the most frequently affected site was medial for the knee (54.7%) and medial, antero- and postero-medial for the elbow (57.3%). These results could be explained by these particular locations having the worse repercussions (e.g., functional, compressive) and therefore needing surgery.

Currently, the only effective treatment for HO is surgery [4], [5], [6], [21]. Indications for surgery have changed recently [4]. In our experience, surgery is indicated when HO causes loss of function, when pain is difficult to manage medically or with risk of nerve or, more rarely, vessel involvement. In our study, the median time from CNS damage to surgery was 15.7 months (range 9.2 to 37.5 months). This delay was significantly shorter than that for patients with SCI (24.1 months, range 13.4–72.4 months). Discomfort is likely to be a problem earlier in conditions other than SCI because of the typical loss of sensation in SCI and the different functional prognosis.

Some studies, with a small sample of patients with HO, have suggested that early surgery does not increase the rate of recurrence [4], [21], [25]. In our survey, recurrence was not associated with delay from CNS damage to surgery, whether considering patients or surgeries. None of the 181 patients who underwent surgery within a year of the CNS damage experienced HO recurrence during follow-up. In addition, 4 patients with recurrence had previously benefitted from surgery for troublesome HO on another location without recurrence. The minimum follow-up by the surgeon in our study was 3 months. In our experience, recurrences requiring surgery appear sooner than 3 months. Furthermore, most patients who underwent surgery for HO came from units in close contact with our institution, and, in cases of recurrence, the surgeon is contacted quickly. Likewise, after 6 months, patients were followed up by a physical and medical rehabilitation physician. Therefore, recurrences were unlikely to be undiagnosed and unreported.

Several other groups have assessed the effect of delayed surgery on HO. Lazarus et al. studied 24 patients who underwent surgery for elbow HO and found that a long delay before surgery was a negative predictor of recovery of ROM [33]. This finding is reinforced by the new approaches in neuro-rehabilitation involving limited ROM. Limited ROM may induce plastic cerebral changes, such as atrophy of motor areas with time, and therefore reduce recovery capacity after ROM has been restored [34]. However, several studies suggested that the more limited the ROM in the pre-operative joint, the better the surgical outcome[33], [35]. The sooner the troublesome HO is treated by surgery, the better the functional outcome. Moreover, in a previous study we found that a long delay before surgery can be deleterious, especially for the ankylosis state: often considerable bone loss of articular structure (i.e., femoral head) and high risk of peri-operative fracture [20]. Because of our large sample size, our results demonstrate that delay is not a criterion to decide surgery, as was suggested by our previous work [20], systematic reviews and other previous studies with limited sample size [4], [6], [25], [26]. Almost half of the patients experiencing recurrence (15 of 31) had multisite HO, so this after-effect might occur more in patients with a global increase of bone activity. However, recurrence was not associated with etiology, sex or age at CNS damage.

According to Garland et al. [36], after SCI, there is a significant relationship between hip HO volume and recurrence risk. Garland proposed a “subjective” radiological grading system in a spinal cord injured group of patients (19 patients with 24 HO). There were 5 grades (from minimal to ankylosis), only for the hip. Previously, Brooker et al. (1973) proposed a radiological scale for post operative patients (after total hip arthroplasty) [37]. There were 4 classes (from “Island of bone within the soft tissues about the hip” to “apparent bone ankylosis of the hip”) and again only for the hip. Stover et al. (1991) proposed to extend this classification for other articulations and aetiologies and mainly after neurological diseases [16]. Finally, Stover suggested (without carrying out a statistic analysis) that the higher the Brooker status, the higher the recurrence risk [16], [37]. Unlike the results of these studies, in our series, the pre-operative extent of HO does not seem to influence the recurrence risk. However, our database is quite varied, containing many aetiologies (4), articulations (4) and surgical indications (functional disabilities, pain, nerve and vessel compressions, hygienic access…) making it difficult to draw reliable conclusions regarding the implication of the volume of preoperative HO in the recurrence risk. Further studies should be carried out on sub-populations of this database (i.e. after TBI and Hip HO). Furthermore, we specify that, in our series, all patients undergoing symptomatic HO were classified as Brooker level 3 or 4 for the hip. As we stated above, we believe the most pertinent risk factors are clinical such as aetiology (SCI), an infectious context (bacteriuria, slough, post operative or post traumatic sepsis such as open fracture), spasticity and the severity of the neurological initial damage.

Our results can be generalized only to patients with HO requiring surgical intervention, not those with HO in general. The prevalence of HO risk is probably underestimated slightly because non-symptomatic cases remain undiagnosed. As well, our study was monocentric, which may influence the generalizability of results. However, our center is a national reference center of rehabilitation, and our large sample size and our patients admitted from a large geographical area should limit questions of recruitment bias.

In conclusion, we performed a large-sample study of HO, a frequent complication after CNS damage but rarely studied, to illuminate features of HO associated with etiology and other factors. In patients with CNS damage, troublesome HO and recurrence occurred most frequently in those with TBI and appeared frequently in the hip and elbow. Early surgery for HO is not a factor of recurrence. Therefore, troublesome HO is the main factor indicating surgery.
